# Antioxidant Therapeutic Strategies for Cardiovascular Conditions Associated with Oxidative Stress

**DOI:** 10.3390/nu9090966

**Published:** 2017-09-01

**Authors:** Jorge G. Farías, Víctor M. Molina, Rodrigo A. Carrasco, Andrea B. Zepeda, Elías Figueroa, Pablo Letelier, Rodrigo L. Castillo

**Affiliations:** 1 Departamento de Ingeniería Química, Facultad de Ingeniería y Ciencias, Universidad de La Frontera, Temuco 4780000, Chile; jorge.farias@ufrontera.cl (J.G.F.); andrea.zepeda.p@gmail.com (A.B.Z.); efigueroavillalobos@gmail.com (E.F.); pablolete@gmail.com (P.L.); 2 Unidad de Cuidados Intensivos, Hospital de Niños Roberto del Río, Santiago 7500922, Chile; victor.molina.cancino@gmail.com; 3 Unidad de Cuidados Intensivos Pediátricos, Hospital Clínico Pontificia Universidad Católica de Chile, Santiago 7500922, Chile; 4 Laboratorio de Investigación Biomédica, Departamento de Medicina Interna, Hospital del Salvador, Santiago 7500922, Chile; r_carrasco_l@yahoo.com; 5 Departamento de Cardiología, Clínica Alemana, Santiago 7500922, Chile; 6 Núcleo de Investigación en Producción Alimentaria, BIOACUI, Escuela de Acuicultura, Universidad Católica de Temuco, Temuco 4780000, Chile; 7 School of Health Sciences, Universidad Católica de Temuco, Temuco 4780000, Chile; 8 Programa de Fisiopatología Oriente, Instituto de Ciencias Biomédicas, Facultad de Medicina, Universidad de Chile, Santiago 7500922, Chile

**Keywords:** hypoxia, oxidative stress, cardiac tissue, microRNA, omega 3 fatty acids, carvedilol, congenital heart disease

## Abstract

Oxidative stress (OS) refers to the imbalance between the generation of reactive oxygen species (ROS) and the ability to scavenge these ROS by endogenous antioxidant systems, where ROS overwhelms the antioxidant capacity. Excessive presence of ROS results in irreversible damage to cell membranes, DNA, and other cellular structures by oxidizing lipids, proteins, and nucleic acids. Oxidative stress plays a crucial role in the pathogenesis of cardiovascular diseases related to hypoxia, cardiotoxicity and ischemia–reperfusion. Here, we describe the participation of OS in the pathophysiology of cardiovascular conditions such as myocardial infarction, anthracycline cardiotoxicity and congenital heart disease. This review focuses on the different clinical events where redox factors and OS are related to cardiovascular pathophysiology, giving to support for novel pharmacological therapies such as omega 3 fatty acids, non-selective betablockers and microRNAs.

## 1. Introduction

Hypoxia-related cardiovascular pathologies, such as myocardial infarction, stroke, peripheral vascular disease and renal ischemia, are among the most frequent causes of death and disability [1]. Hypoxia is defined as the threshold where the oxygen concentration is a limiting factor for normal cellular processes, including ATP synthesis. The integration of local responses defines hypoxia as a paradigm of reactions affecting the entire body [2]. Subsequently, an oxygen gradient arises between affected and non-affected tissues, stimulating the migration and proliferation of endothelial cells and fibroblasts, thereby reconstituting normal oxygen supply by increasing perfusion [3]. If this process fails, a prolonged inadequate vascular supply of oxygen leads to chronic hypoxia and can cause chronic diseases. Conversely, some cardiovascular diseases are related to the re-exposure to physiologic or supra-normal oxygen concentrations after a hypoxic insult, which constitute the basis for ischemia–reperfusion injury. Oxidative stress (OS) seems to be a common pathway in several morbid states in which myocardial injury is the primary determinant. In this review, the involvement of oxidative stress in cardiovascular disease is explored and redox-based strategies are reviewed in representative conditions that serve as prototypical models for antioxidant therapies development.

## 2. Oxidative Stress in Cardiovascular Disease

For decades, oxidative stress (OS) was defined as an imbalance between the production of reactive oxygen species (ROS) and antioxidant defenses in the cell, which leads to oxidative damage of cell structures, including lipids, membranes, proteins and DNA [4]. This process results in inactivation of essential metabolic enzymes and disruption of signal transduction pathways [5]. Now it is clear that differences in subcellular and tissue compartmentalization of ROS contribute to stress responses [6]. It is important to know that ROS is produced as a result of normal cellular metabolism processes [7], while antioxidants eliminate oxidants and repair the damage caused by ROS [8]. Intracellular oxidative stress is produced in normal conditions by the formation of ROS as the result of normal mitochondrial respiration, but also during reperfusion in hypoxic tissue and in association with infection and inflammation [9]. ROS overproduction has been implicated in endothelial injury and extracellular/intracellular OS [10]. Additionally, OS has been implicated in a wide array of diseases such as neurodegenerative disorders, autoimmune diseases, complex lifestyle diseases and cancer [5], and it is central in the pathogenesis of more than 100 inflammatory disorders like periodontitis, diabetes, rheumatoid arthritis, stroke and inflammatory lung diseases [11,12,13,14].

### Sources of ROS in Cardiovascular Pathologies

Reactive oxygen species is a collective common term that includes highly oxidative radicals such as hydroxyl (OH-) and superoxide (O_2_^•−^) radicals, and non-radical species such as hydrogen peroxide (H_2_O_2_). The term can also include reactive nitrogen species, and both species are normal metabolism byproducts [15,16]. Low concentrations of ROS are required for many cellular processes, whereas overproduction is controlled and/or ameliorated by antioxidants [17].

Mitochondria are a major source for intracellular ROS generation. Within the electron transport chain, a premature leak of a small percentage of electrons to oxygen results in physiological ROS production. Antioxidants in the mitochondria such as superoxide dismutase (SOD)-2 and glutathione rapidly degrade or sequester O_2_^•−^ to reduce reactivity. Perhaps due to high concentrations of mitochondria in cardiac tissue, reduced mitochondrial antioxidant capacity results in cardiac dysfunction [18]. Accordingly, mitochondrial damage or dysfunction results in mitochondrial cellular oxidative stress [19].

In addition, ROS have been involved in a wide range of vascular diseases associated with the functional properties of the endothelial cell barrier [20]. It has been proven that oxidized low-density lipoprotein (ox-LDL) increases ROS formation in human umbilical vein endothelial cells (HUVECs) through association with a specific endothelial receptor, which may trigger nuclear factor-κB (NF-κB) activation to induce ROS formation [21]. Among other known sources that increase ROS levels are angiotensin II and uremic toxin indoxyl sulfate-induced endothelial cell dysfunction [22]. Glucose can generate ROS via various pathways including mitochondria, nicotinamide adenine dinucleotide phosphate (NADPH)-oxidase, the sorbitol pathway, activated glycation and the insulin pathway, suggesting that sugars are involved in the development of atherosclerosis, hypertension, peripheral vascular disease, coronary artery disease, cardiomyopathy, heart failure and cardiac arrhythmias, and that these effects of added sugars are mediated through ROS [23]. NADPH oxidase (Nox) signaling is essential for normal physiology, but upregulated and overactive Nox enzymes contribute to oxidative stress and cardiovascular disease [24]. Enzymes of the lipoxygenase (Lox) family catalyze the oxidation of polyunsaturated fatty acids, and increased expression of 5-lipoxygenase (5-LO) has been found in atherosclerotic plaque and abdominal aortic aneurysms [25]. Myeloperoxidase (MPO) is a heme-containing peroxidase expressed in neutrophils and monocytes, and it is believed to produce ROS that contribute to lipid oxidation in atherosclerosis [26].

## 3. Cardiac Diseases Related to Myocardial Hypoxia/Ischemia

Cardiac hypoxia is the result of a disproportion between oxygen supply and demand. Due to high coronary arteriovenous differences, the myocardium is unable to bring about substantial improvements in oxygen supply by increasing oxygen extraction from the blood. Thus, the only way to meet the higher oxygen demand is by increasing the blood supply. Theoretically, any of the known mechanisms leading to tissue hypoxia could be responsible for a reduction in oxygen supply in the cardiac tissue. However, the most common causes are undoubtedly: (1) ischemic hypoxia (often described as “cardiac ischemia”) induced by the reduction or interruption of the coronary blood flow; and (2) systemic (hypoxic) hypoxia (“cardiac hypoxia”) characterized by a fall in PO_2_ levels in the arterial blood, but with adequate perfusion [27,28]. The effects of ischemia are usually more severe than hypoxia and typically include lactic acidosis due to anaerobic glycolysis, impaired mitochondrial energy production, and cell death [29].

Hypoxia is a major contributor to cardiac pathophysiology, including myocardial infarction, cyanotic congenital heart disease and chronic cor pulmonale [30]. A chronic lack of oxygen leads to an increase in pulmonary vasoconstriction that redistributes pulmonary blood flow from low to high PO_2_ regions [31]. However, chronic pulmonary vasoconstriction may result in pulmonary hypertension, increasing the afterload on the right ventricle, which may eventually lead to heart failure.

### 3.1. Therapeutic Strategies Related with Hypoxia/Ischemia

The most effective treatment to reduce infarct size following hypoxic insult is the re-opening of the culprit-occluded coronary artery by coronary angioplasty or thrombolysis. Adjunctive treatments at reperfusion, such as β-blockers and angiotensin-converting enzyme inhibitors, can ameliorate morbidity and mortality, although not via a reduction in infarct size [32]. Despite these improvements in treatment, mortality remains elevated in high-risk patients and the prevalence of heart failure is also increasing [33], which justifies the search for therapies that would effectively reduce infarct size. These strategies that relate the control of the magnitude and time of tissue hypoxia to functional/structural outcome (e.g., left ventricular function) can be separated into pharmacological or non-pharmacological strategies.

#### 3.1.1. Cardiac Ischemic Preconditioning

Cardiac ischemic preconditioning was first described in 1986 in dogs subjected to brief, intermittent episodes of ischemia. These episodes had a protective effect on the myocardium that was later subjected to a sustained ischemia cycle [34]. This protocol revealed that infarct size was reduced by 75% in dogs exposed to intermittent occlusion of the circumflex artery for 4–5 min followed by 40 min of total occlusion. Accordingly, brief ischemia of the myocardium initiated a cascade of biochemical events in cardiomyocytes that protect the heart muscle during subsequent ischemic insults [35]. The mechanisms underlying these endogenous cardioprotective phenomena are complex in nature and are conventionally divided into triggers, mediators and effectors. Signaling pathways involve surface receptors such as adenosine, bradykinin and opioid-signaling kinases (for example PI3K-Akt-eNOS, JAK–STAT3, PKC, among others) and mitochondrial components (mitochondrial potassium channel dependent on ATP (mKATP), mitochondrial permeability transition pore (mPTP) and protein kinase C) [36]. The cellular and paracrine effects of preconditioning in cardiomyocytes include the induction of angiogenesis and progenitors, stem cell activation and the attenuation of cell death, inflammation, and adverse remodeling [37]. Because classic preconditioning must be implemented before the onset of severe myocardial ischemia, its clinical use is largely restricted to specific situations, such as cardiac surgery, in which the ischemic injury can be anticipated. The non-genetic approach of ischemic preconditioning to enhance cell- and tissue-based therapies has received a great deal of attention in recent years due to its non-invasive, drug-free application. Therefore, the use of pharmacological preconditioning strategies to obtain cardioprotection through classic cellular targets and studies on new targets are currently in development. Clinical use of the ischemic approach has been controversial to date. However, the current design of a reperfusion intervention has provided the basis for new therapy in cardiovascular pharmacology against ischemia–reperfusion injury [38].

#### 3.1.2. Pharmacological Preconditioning

The discovery of ischemic preconditioning has shown the existence of intrinsic systems of cytoprotection, the activation of which can stave off the progression of irreversible tissue damage. Deciphering the molecular mediators that underlie the cytoprotective effects of preconditioning can pave the way to important therapeutic possibilities. Pharmacological activation of critical mediators of ischemic preconditioning would be expected to emulate or even intensify its salubrious effects. Indeed, it is possible to avoid the detrimental effects of ischemia as a maneuver of cardioprotection [36].

Extensive experimental research carried over the past two decades to elucidate the complex signaling pathways underlying the cardioprotective effects of ‘conditioning’ therapies have led to the discovery of several pharmacological agents able to reproduce the benefits of these mechanical procedures [39]; hence, the term pharmacological preconditioning has been introduced. For example, anesthetic drugs have been shown to possess such properties and, therefore, allowed for anesthetic preconditioning [40]. These denominations refer to cardioprotection triggered by anesthetics administered in this setting (i.e., before prolonged ischemia). In the case of volatile anesthetic conditioning, it is considered less risky and safer in clinical application than its ischemic counterparts, particularly in the diseased myocardium because it does not require the direct administration of the therapeutic agent into the coronary artery; moreover, after inhalation, it provides systemic protection [41]. In the context of protection of the myocardium, nervous system, gut, and kidney beyond the duration of exposure to the volatile agent, anesthetic conditioning may offer additional benefits during the critical postoperative period and may also have a direct impact on long-term prognosis and clinical outcome [42]. Cardioprotective effects of anesthetic agents depend on the interaction of various factors such as administration protocols, choice of specific agents, concomitant use of other drugs, and the variables used to assess myocardial function. There are many confounding factors that limit the applicability of anesthetic conditioning in humans. Some issues to be resolved by future experimental studies are: gender differences, maximum duration of index ischemia, optimal duration and concentration of drug, and potential interactions with other drugs [38].

**Figure 1:**
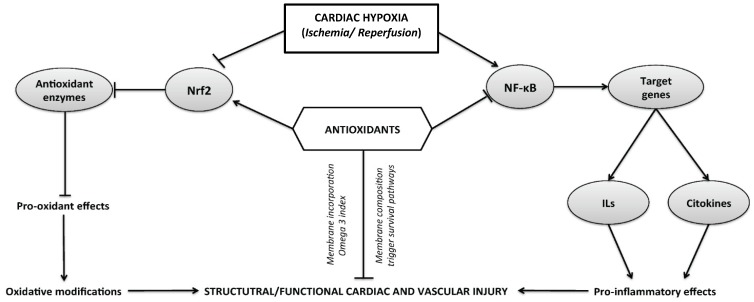
Representation of the cellular and molecular pathways of damage induced following a time course of hypoxia and ischemia–reperfusion cycle in different tissues. The activation of enzymatic and non-enzymatic sources of reactive oxygen species (ROS) is associated with the modulation of redox-sensitive transcriptional factors, such as the activation of nuclear factor (NF)-κB and inhibition of nuclear factor erythroid 2—related factor 2 (Nrf2). Both cellular pathways are implicated in the oxidative modifications or pro-inflammatory effects that can mediate structural or functional cardiovascular impairment.

The mechanistic evidence that supports the cardioprotective effects of antioxidants, and the molecular mechanisms triggered at cardiac and vascular tissue are shown in Figure 1.

### 3.2. Novel Antioxidant-Based Therapies in Ischemia–Reperfusion

#### 3.2.1. Cardiac Preconditioning with Omega 3

In vitro studies, animal experiments, observational studies and randomized clinical trials have examined the cardiovascular effects of seafood consumption and long-chain omega-3 polyunsaturated fatty acids. These types of fatty acids are composed of eicosapentaenoic acid (EPA; 20:5 *n*-3), docosahexaenoic acid (DHA; 22:6 *n*-3) and α-linolenic acid (ALA; 18:3 *n*-3). Alpha-linolenic acid is a plant-derived omega 3 found in a relatively limited set of seeds, nuts and their oils. Alpha-linolenic acid cannot be synthesized in humans and it is an essential dietary fatty acid [43]. There are biochemical pathways to convert ALA to EPA and EPA to DHA, but such endogenous conversion is limited in humans: between 0.2% and 8% of ALA is converted to EPA (with conversion generally higher in women) and 0% to 4% of ALA to DHA (10–14). Thus, tissue and circulating EPA and DHA levels are primarily determined by their direct dietary consumption [44]. Recent studies suggest that the beneficial effects of fish oil are due, in part, to the generation of various free radical-generated non-enzymatic bioactive oxidation products from omega 3, although the specific molecular species responsible for these effects have not been identified. It is of interest to note that the beneficial effects of EPA and DHA could arise from both direct short-term or long-term effects mediated by changes in some intracellular pathways as discussed below. Direct actions of omega 3 have been confirmed by experimental studies of sudden cardiac death in a reliable dog model, showing that these compounds electrically stabilize heart cell membranes through the modulation of the fast voltage-dependent Na^+^ currents and the L type Ca^2+^ channels. Derived from this effect, cardiac cells become resistant to arrhythmias [45,46]. Moreover, it has been pointed out that *n*-3 polyunsaturated fatty acids (PUFA) can exert a reversible modulation in the kinetics of several ion channels by binding to specific sites on channel proteins and by non-specifically incorporating them into lipid cell membranes [47]. These changes are consistent with the type of fatty acids incorporated into the cardiac tissue membrane.

With regard to diet supplementation, it has been noted that these rich in omega-3 polyunsaturated fatty acids are associated with decreased incidences of cardiovascular disease. The extent to which these beneficial fats are incorporated into and distributed throughout body tissues is uncertain. In some animals supplemented for more than two weeks with diets enriched with omega 3 as fish oil, the incorporation kinetics of both EPA and DHA have been measured, and this might be associated with the tissue response profile to an injury [48]. In the case of the heart and blood vessels, this would determine the type of hemodynamic response to a pro-inflammatory and pro-oxidant injury. For example, controlled ischemia in an ex vivo model may induce a greater recovery of ventricular function if the supplementation has a high incorporation of DHA in the cardiac tissue [1,49]. These kinetics would also allow a more efficient anti-oxidant and anti-inflammatory response at the heart tissue level [50,51]. However, consumption of dietary flaxseed appears to be an effective means to increase ALA content in body tissues, but the degree will depend upon the tissues examined.

In relation to chronic consumption, it has been reported that omega 3 is selectively incorporated into cardiac cell membranes in a dose-related manner after 8 weeks of supplementation [52]. Also, omega 3 can improve post-ischemic functional recovery in the ex vivo Langendorff perfusion of rat heart, also suggesting the benefit of a diet highly enriched with omega 3 content [53,54]. Regular intake can slow the heart rate, reduce myocardial oxygen consumption, and increase coronary reserve. These properties contribute to preconditioning-like effects of resistance to myocardial infarction and improved post-hypoxic recovery. These effects can be demonstrated in isolated hearts, regardless of the effects of omega 3 on neural or blood parameters. Also, the enrichment of myocardial membranes with omega 3 reduces vulnerability to cardiac arrhythmias, particularly ventricular fibrillation, and attenuates heart failure and cardiac hypertrophy [55].

#### 3.2.2. Antioxidant Mechanism Induced by Omega 3

Experimental evidence demonstrates that antioxidant effects of omega 3 are related to the incorporation of these compounds into the cell membrane and the modulation of redox signaling pathways. In this view, omega 3 supplementation increases the expression and activity of the antioxidants enzymes and attenuates thiobarbituric acid-reactive substances (TBARS) increased in rats ([56], Erdogan et al., 2004). Oxidized omega 3 reacts directly with Keap1, a negative regulator of Nrf2, initiating Keap1 dissociation with Cullin3 and thereby inducing Nrf2-dependent target antioxidant genes such as heme oxygenase-1 ([57], Gao et al., 2007). This omega 3-antioxidant reinforcement is associated with a reduction in the susceptibility of myocytes to ROS-induced IR injury, and to an increase in SOD and GSH-Px expressions ([46], Jahangiri et al., 2006). Animal studies showed that the cardioprotective effects of PUFA can be exerted through the upregulation of heat shock protein 72, a key preconditioning protein, and higher omega 3 content of myocardial membranes, which appears to facilitate the protective response to hypoxic injury ([58], McGuinness et al., 2006). Recently, hearts supplemented with omega 3 showed lower infarct size and a higher left ventricular pressure compared with non-supplemented rats. Hearts in the supplemented group with omega 3 showed lower levels of oxidative stress markers and higher antioxidant activity, decreased activity and NF-κB and Nrf2 activation, compared with the non-supplemented group.

#### 3.2.3. Microribonucleic Acids (miRNAs)

Hypoxia is a powerful stimulus, regulating the expression of a specific subset of microribonucleic acids (miRNAs), called hypoxia-associated miRNA (hypoxamiRs). Accordingly, several hypoxamiRs are involved in cardiac development and ischemic cardiovascular diseases [59,60]. miR-210 represents major hypoxia-inducible miRNAs, and has been studied for its effects such as promotion of cell survival and improvement of heart function [61], possibly via the upregulation of angiogenesis and inhibition of cardiomyocyte apoptosis [62]. Moreover, evidence indicates that some miRNAs may have a direct (synergistic) effect on the expression of the hypoxia-inducible factor (HIF), a key regulator of the transcriptional response to hypoxia, and thus a negative-feedback loop in cells exposed to prolonged hypoxia [63]. On the other hand, the vascular endothelial growth factor (VEGF) and HIF-VEGF pathways are related to the pathophysiology of ischemic vascular disease [64]. These pathways can also be regulated epigenetically by miRNAs. Interestingly, some of these miRNAs, such as miR-206, significantly suppress the viability and invasion and promote the apoptosis of endothelial progenitor cells in coronary artery disease patients by modulating VEGF expression [64]. Accumulating evidence suggests that miRNAs have antiangiogenic properties, for example, miR20b modulates HIF-1a, STAT3 and VEGF expression [65,66], mir-221 and miR-222 target c-kit and eNOS [65,66,67], miR-320 targets insulin-like growth factor (IGF)-1 in the diabetic endothelial cells [68], and miR-145 reduces microvascular cell migration in vitro [69]. On the other hand, some pro-angiogenic factors have been described, such as mir-296 [70] miR-130a [71], miR-126 [72,73,74], miR-210 [75,76], let-7f and miR-27b [77].

##### miRNA as a Therapeutic Strategy

miRNAs are new and powerful candidates for therapeutic intervention against various pathological conditions, including cardiovascular diseases [78]. miR-1 is the most abundant miRNA specific to cardiac and skeletal muscle [79,80]. It is an important regulator of cardiomyocyte growth in the adult heart as well as a pro-apoptotic factor in myocardial ischemia [81], relating to diseases such as hypertrophy, myocardial infarction and arrhythmias [82,83], and can be used as a myocardial infarction biomarker [84]. It has been demonstrated that increased miR-1 levels significantly reduce infarction size [85]. In addition, intracardiac injection of miR-21 along with miR-1 and miR-24 was reported to reduce infarct size in a rat model [81]. The transplantation of mesenchymal stem cell (MSC) delivery overexpressing miR-210 and miR-1 to the infarcted rat hearts improved cardiac function [86]. By contrast, repressed miRNAs have a protective effect, such as the inhibition of microRNA-377 function by antagomir transplantation of MSC. It was observed to reduce fibrosis and improve myocardial function [87], and anti-miR-29 antagomirs significantly reduced myocardial infarct size [88].

Some studies with natural compounds indicate that miRNA expression is modified following administration of antioxidant compounds, functioning as a protective mechanism for cardiovascular disease. For example, miR-126 expression was increased in colon-derived myofibroblast cells upon treatment with wine-derived polyphenols, and that response was associated with a reduced expression of inflammatory genes [89]. Also, therapy with luteolin-7-diglucuronide (L7DG), a naturally-occurring antioxidant found in edible plants, attenuated altered isoproterenol (ISO) expression of miRNAs associated with induced myocardium injury and fibrosis in mice [90]. In addition, H_2_O_2_ modifies the expression of microRNAs ([91], Wei, Gan et al., 2016). These studies show that miRNAs are modulated by antioxidant compounds and ROS, however they also can be involved in ROS production. For example miR-135a participates in the regulation of H_2_O_2_-mediated apoptosis in embryonic rat cardiac myoblast cell lines ([92], Liu, Shi et al., 2017]). A study in an animal model revealed that miR-133a via targeting uncoupling protein 2 (UCP2) participates in inflammatory bowel disease by altering downstream inflammation, oxidative stress and markers of energy metabolism ([93], Jin, Chen et al., 2017). The silencing of miR-155 modulated stress oxidatives by decreased ROS and promoted nitric oxide (NO) generation in human brain microvessel endothelial cells (HBMECs) via regulating diverse gene expression (caspase-3, ICAM-1, EGFR/ERK/p38 MAPK and PI3K/Akt pathways) ([94], Liu, Pan et al., 2015). The overexpression of miR-103 abrogated cell activity and ROS production induced by H_2_O_2_, via targeting Bcl2/adenovirus E1B 19 kDa interacting protein 3 (BNIP3) in HUVEC cell lines ([95], Xu, Gao et al., 2015). These studies provide novel clues and potential future therapeutic targets for the treatment cardiovascular disease.

The possible future clinical applications include the use of different strategies based on inducing or repressing miRNA expression [96]. For repression, antagonists (antagomirs) inhibit the activity of specific miRNAs. In contrast, miRNA mimics are used to restore miRNAs that show a loss of function [96,97]. However, one of the major obstacles of this therapy is low stability and bioavailability. Small molecules (less than 50 kDa) are generally filtered by the kidney and subsequently excreted. Furthermore, macrophages and monocytes rapidly remove RNA complexes from the circulatory system. For this reason, different modifications have been generated in microRNAs, which improve the availability and the effect in vivo [97] Also, several strategies (delivery vehicles for miRNA therapeutics) have been used to improve their bioavailability, such as liposomes, polymers (the cationic polymer polyethylene imine), conjugates, exosomes and bacteriophages [98]. miR-122 was the first miRNA that underwent successful clinical trials in hepatitis C virus (HCV)-infected patients [96] and it is a unique miRNA with therapeutic potential both as an anti-mir in combating HCV infection and as a mimic against various liver diseases [99]. Other clinical studies are being carried out, mainly in oncological disease, where the partial responses observed are very promising [100]. In the cardiovascular field, promising preclinical studies suggest that miRNAs could be useful in treating these disorders, although several challenges related to specificity and targeted delivery remain to be overcome.

## 4. Oxidative Stress and Cardiotoxicity

As an inclusive definition, cardiotoxicity is the development of myocardial injury as a response to an endogenous or exogenous agent. Several forms of cardiotoxicity include oxidative stress among their pathophysiological mechanisms, such as cardiotoxicity in takotsubo cardiomyopathy, cocaine-mediated cardiotoxicity, sepsis-induced myocardial dysfunction, and others [101,102,103]. Oxidative stress also counts among the mechanisms of cardiotoxicity of some pharmacological treatments in the context of adverse effects, where chemotherapeutic drugs have been studied the most [104].

### 4.1. Chemotherapy-Induced Cardiotoxicity Secondary to the Collateral Damage of Oxidative Stress on Non-Target Tissues

Injury caused by chemotherapy in non-target tissues often complicates cancer treatments by limiting the use of optimal therapeutic doses of anticancer drugs and impairing the quality of life of patients during and after treatment. Oxidative stress, directly or indirectly caused by chemotherapeutics, is one of the underlying mechanisms of the toxicity of anticancer drugs in non-cancerous tissues, with the effects on the heart being the most studied for their great impact on the survival prognosis of these patients [105]. Many of the most commonly used chemotherapy drugs have been reported to induce oxidative stress, including anthracyclines, cyclophosphamide, cisplatin, busulfan, mitomycin, fluorouracil, cytarabine, and bleomycin [105]. In an exceptional way, some of these chemotherapeutic agents, such as bleomycin, could potentially use the generation of oxidative stress as a mechanism for killing cancer cells [106]. In the vast majority of chemotherapeutic agents the generation of oxidative stress has no role in antineoplastic effectiveness and the induction of oxidative stress occurs in non-target tissues and thereby leads to “normal tissue injury” [105]. In addition, some of the new molecularly targeted therapies in oncology may also induce oxidative stress, such as trastuzumab [107]. Trastuzumab, a monoclonal antibody against ErbB-2 (HER2), induces cardiac dysfunction through the alteration of NADPH oxidase and mitogen-activated protein kinase (MAPK) signaling pathways [108,109]. Furthermore, this alteration of HER2 signaling through NADPH oxidase and MAPKs has been associated with an increase in oxidative stress, leading to dilated cardiomyopathy [109,110].

Thus, although the use of classical chemotherapeutic agents with new molecularly targeted treatments has greatly improved survival rates, leading in some cases to curing the cancer, the oxidative stress-mediated impairment of normal tissues is a significant side effect and decreases patients’ quality of life [105], with particular relevance to the cardiovascular effects, since these are the ones that most determine the prognosis of these patients. A better understanding of the mechanisms involved in oxidative heart injury is essential to the design of intervention strategies that will attenuate the cardiotoxicity of chemotherapeutic agents without compromising their anticancer efficacy.

### 4.2. Mechanisms of Anthracycline-Induced Cardiotoxicity

Anthracyclines tend to accumulate in the mitochondria, which explain their predilection for myocardial tissue that has a high mitochondrial density due to its high metabolic demand [111]. The classic anthracycline cardiotoxicity hypothesis proposed that ROS generation is the initial event that leads to redox imbalance [112]. ROS generation may be due to the anthracycline effects on complex I of the electron transport chain, after reduction of anthracycline ring C, leading to the formation of the free radical semiquinone [113,114]. This radical is relatively stable in an anoxic environment medium, but in normoxic conditions, the unpaired electron is donated to the oxygen, forming superoxide radicals. Complex I, through flavoproteins, catalyzes the formation of the reduced semiquinone radical, first accepting electrons from NADH or NADPH, and then delivering them to anthracyclines. This sequence of reactions is known as “redox cycling” and may be highly detrimental because a relatively small number of anthracyclines is sufficient for the formation of numerous superoxide radicals with the ensuing oxidative injury [115,116].

However, in recent years a new hypothesis has focused on “Top2β” as the initial event of cardiotoxicity [117]. In this sense, from a pathophysiological point of view, this new hypothesis has displaced the generation of reactive oxygen species as the first initiator event at an early stage of damage, putting ROS generation at a later stage or as a downstream event, being a consequence of the alterations produced by the interaction between anthracyclines and Top2β.

Whatever the case, either as an initiating or a downstream event, oxidative stress emerges as an attractive target to prevent cardiotoxicity with antioxidant therapies without compromising the anticancer effectiveness of anthracyclines.

### 4.3. Preventive Therapies for Anthracycline-Induced Cardiotoxicity with Direct or Indirect Antioxidant Effects

#### 4.3.1. Reactive Oxygen Species Scavengers

Several compounds with antioxidant properties have been studied in vitro with some degree of success [118,119]. Also, in addition to preventing direct damage by oxidative stress, the use of antioxidants could indirectly block the induction of ROS-induced apoptosis. However, although these previous in vitro studies with antioxidants and free radical scavengers have shown an inhibition of myocardial apoptosis [120,121], the success of these interventions in in vivo studies has been less satisfactory. In fact, molecules with antioxidant characteristics such as vitamin E and selenium or nimesulide that had shown good results in vitro showed a poor preventive effect of anthracycline-induced cardiotoxicity in in vivo models [122,123].

This dissociation between the effectiveness of the interventions in vitro and in vivo could be due to the fact that the concentrations of antioxidants that should be reached in myocardial tissue to prevent damage are too high [124]. It is known that the dose indicated to obtain an effective action to eliminate free radicals with vitamin E and C at the myocardial level cannot be obtained with oral contributions; therefore, the findings of a null clinical action of therapies provided by this route are highly predictable.

#### 4.3.2. Prevention of Reactive Oxygen Species Generation

Another antioxidant strategy focused on preventing the generation of ROS may seem more effective than the classic interventions with antioxidant free radical scavengers. In this sense, most of the cardiotoxicity preventive strategies with antioxidant effects currently under study are characterized by their potential mechanism of action being directed toward mitochondrial ROS generation.

##### Carvedilol

Carvedilol, a competitive blocker of β1, β2 and α1-adrenergic receptors, is widely used clinically for the treatment of heart failure, hypertension and acute myocardial infarction.

One distinctive feature of carvedilol is a potent antioxidant property, which is not shared by other β-adrenergic receptor antagonists [125]. The observation that carvedilol also acts as an inhibitor of mitochondrial complex-I is of importance, since this mitochondrial system was proposed to be involved in the mechanisms of anthracycline-induced cardiotoxicity [126]. Carvedilol is therefore superior to other beta blockers, such as atenolol, in reducing the negative impact induced by doxorubicin on the left ventricular ejection fraction, as well as increased lipoperoxidation in in vivo models [127]. This has been further confirmed by other in vivo studies in which carvedilol decreased both mitochondrial and histopathological cardiac toxicity caused by anthracyclines [128].

A clinical study evaluated the cardioprotective role of carvedilol against the cardiotoxic effect of anthracyclines, determining that the preventive use of carvedilol allowed the preservation of left ventricular systolic function at six months, based on echocardiographic observation variables [129]. Another study, the OVERCOME Trial (prevention of left ventricular dysfunction with enalapril and carvedilol in patients undergoing intensive chemotherapy for the treatment of malignant hemopathies), a randomized clinical trial evaluating a combined treatment of enalapril and carvedilol, was able to prevent reduction in the left ventricular ejection fraction in hemato-oncologic patients who had received intensive chemotherapy. This was an encouraging strategy that should be confirmed by larger clinical trials [130].

##### Omega-3

Omega-3 represents an attractive preventive strategy due its ability to reduce the susceptibility to oxidative stress injury in myocardial cells. This effect is explained by previously discussed mechanisms, such as increased antioxidant defenses, changes in membrane fluidity and the ability to prevent the release of intracellular calcium in response to oxidative stress [44].

The first clinical studies with omega-3 in oncology patients were aimed at improving the antineoplastic effect of chemotherapy. A study in breast cancer patients receiving anthracycline chemotherapy, which used DHA to improve sensitivity to chemotherapy, found no major adverse side effects [131]. Subsequently, other studies that used omega-3 before or during chemotherapy were able to improve the effectiveness of the chemotherapy [132]. In relation to the potential benefit of a non-ischemic preconditioning that could be offered with the use of omega-3, several animal studies have evaluated the effectiveness of preconditioning as a cardioprotection mechanism against anthracycline-induced cardiotoxicity. It has been established that ischemic preconditioning decreases the cardiotoxicity due to anthracycline, assessed with echocardiographic control of left ventricular function. This type of cardioprotection can also ameliorate the apoptosis rate in cardiomyocytes [133,134,135].

Specifically, there are not many studies that have evaluated the effect of omega-3 to prevent anthracycline-induced cardiotoxicity. Among the few studies available, an animal model study found that omega-3 did not increase anthracycline cardiotoxicity, which contrasted with a study by Carbone et al., where omega-3 not only failed to prevent cardiotoxicity, but exacerbated anthracycline cardiotoxicity [136,137]. This paradoxical situation has not been evidenced in clinical studies in cancer patients who have used omega-3. This could be explained by the fact that this study was carried out on a sheep model, which involves a totally different metabolic model of the fatty acids compared to human metabolism, because this is an herbivorous animal model.

Finally, a study carried out on rats, a metabolic model more similar to humans with respect to fatty acids, evaluated the cardiac effect of omega-3 on the function and histology in a model of anthracycline heart failure. In this study, the diet with omega-3 supplementation attenuated anthracycline-induced cardiac dysfunction, suggesting that this might be associated with an earlier recovery of cytokine imbalance caused by anthracyclines [138].

### 4.4. Chemotherapy-Induced Cardiotoxicity of Other Non-Anthracycline Agents

The non-anthracyclines agents may have multiple manifestations of cardiovascular toxicity, including left ventricular dysfunction, hypertension, ischemia and QT prolongation [139]. In addition, its cumulative incidence can be high, as for example, heart failure at 10 years is 32.5% after non-anthracycline chemotherapy regimens, therefore it also represents an important impact on surviving chemotherapy patients [139]. Although the antineoplastic mechanisms of action of these chemotherapeutics agents are multiple, they have in common the induction of oxidative stress in non-target tissues, thereby leading to “normal tissue injury” [140], which occurs for example with cyclophosphamide, cisplatin, busulfan, mitomycin, fluorouracil, cytarabine, and bleomycin [140].Among these agents, 5-fluorouracil and its prodrug capecitabine are some of the most important agents because they are the most common causes of chemotherapy-related cardiotoxicity after the anthracyclines, and depending on the study, its rates of toxicity range from 1 to 19% [141].

The influence of 5-FU treatment on the antioxidant system in myocardial tissue was studied by Durak et al. [142], They found lowered activities of superoxide dismutase and glutathione peroxidase accompanied by higher catalase activity in 5-FU-treated female guinea pigs. The antioxidant potential, defined relative to malondialdehyde (MDA) levels, declined in 5-FU-treated animals compared with controls, while MDA levels increased [142]. However, the role of oxidative stress in the pathogenesis of 5-FU cardiotoxicity is not well-established, and the source of ROS formation remains undefined. In vitro studies of free radical formation and animal studies investigating the role of iron-chelators may confirm or disprove this hypothesis [143].

More important clinical trials with antioxidant therapies in cardiovascular pathologies related with oxidative stress are shown in Table 1.

## 5. Antioxidant-Based Strategies in Congenital Heart Disease Surgical Correction

Cardiopulmonary bypass (CPB) is known to be associated with postoperative organ dysfunction and with a systemic inflammatory response [147]. Oxidative stress is believed to participate in the pathogenesis of this response, thereby being a potential therapeutic target [148,149]. Major inflammation triggers in these patients include blood–CPB circuit contact, translocation of intestinal endotoxin and myocardial ischemia–reperfusion injury, and also surgical trauma, hypothermia and hemolysis [147]. The contact of blood with the cardiopulmonary circuit elicits an inflammatory response that includes neutrophil activation and superoxide production [150] through the well-known NADPH oxidase-mediated oxidative burst.

The patient’s ability to withstand the inflammatory and oxidative insult depends on the balance between the magnitude of the pro-inflammatory and pro-oxidative insult and the anti-inflammatory and anti-oxidative response, in addition of course to the previous organ function and comorbidities. In this regard, children, and especially newborns, are a particularly vulnerable population due to distinctive characteristics of congenital heart surgery: (1) longer CPB and circulatory arrest duration; (2) greater CPB circuit surface area/patient size ratio; (3) low antioxidant reserve in patients with cyanotic heart defects that will be abruptly re-oxygenated [151,152]; and (4) reduced antioxidant defenses and higher levels of free iron in newborns and especially in pre-term infants [153]. Indeed, in children the reduction in antioxidant defenses during CPB, measured as the total blood glutathione concentration, is inversely related to the CPB duration, and the resulting lipid peroxidation does not return to normal values at 24 h postoperatively [154]. Temporal analysis of oxidative stress biomarkers in children shows that a reduction of plasma ascorbate levels, an increase in its oxidation product (dehydroascorbic acid) and an increase in plasmatic MDA concentration occur early after cross-clamp removal. This study also showed that peak concentrations of IL-6 and IL-8 occur later (3-12 h post-CPB), and that the loss of ascorbate and cytokine concentration correlates with CPB time [155].

Besides systemic oxidative stress, surgery-related myocardial injury in infants with congenital heart disease is of foremost importance, because these hearts almost never have a normal myocardial function and an absolutely normal anatomy is almost never achieved. In patients under 1 year of age undergoing surgical reparation of ventricular septal defect (VSD) or tetralogy of Fallot (TOF), an increase of TBARS, 8-isoprostane and protein carbonyl concentrations in coronary sinus blood after 1–3–5–10 min following aortic cross-clamp removal has been observed [156]. Accordingly, histopathological analysis of the myocardium in infants dying from heart failure after cardiac surgery show ischemic lesions that colocalize with the expression of 4-hydroxynonenal, a lipid peroxidation marker, which may imply a role of oxidative injury in the pathogenesis of these lesions [157].

Despite the abundant evidence showing the effect of CPB on redox balance, the implications of oxidative stress in the clinical outcome of these children is less clear. In a study that compared children after heart surgery with and without low cardiac output syndrome, no differences were found between these two groups in TBARS and carbonyl serum levels in peripheral blood [158]. This study, however, was very heterogeneous in the types of congenital heart malformations that were included. Also, the use of peripheral blood is a limitation when assessing myocardial oxidative damage. By contrast, children undergoing stage II univentricular staging surgery have increased plasma F_2_-isoprostane concentration after CPB that associates with decreased lung compliance, higher PCO_2_ and lower pH, which may imply a role of oxidative stress in postoperative behavior in this specific patient subset [159].

Several oxidative stress therapeutic strategies have been studied in these patients:

### 5.1. Glucocorticoids

Glucocorticoids have been widely used in the past as a way of controlling the inflammatory response, but no clear benefit has been proven [160].

### 5.2. Antioxidants

Although several antioxidant-based strategies in adults have been evaluated, such studies in children are almost non-existent. A small study evaluated the effect of allopurinol supplementation in TOF surgery, showing less ROS expression in myocardial tissue, but no difference in MDA concentration in coronary sinus blood was observed [161]. Also, the use of curcumin, a potent ROS scavenger, in TOF surgery results in decreased c-Jun N-terminal kinase activity in cardiomyocyte nuclei and less caspase-3 expression, which relates to better right and left ventricle systolic function [162].

### 5.3. Controlling Oxygen Supply

The use of normoxic instead of hyperoxic CPB in patients with cyanotic heart disease undergoing surgery results in lower plasma troponin I, lower F_2_-isoprostane concentration, lower protein S100 release (a marker of cerebral injury) and lower alpha-glutamate transferase release (a marker of hepatic injury) [163]. In addition, controlled re-oxygenation in CPB, instead of standard/hyperoxic CPB, results in lower troponin I, F_2_-isoprostanes, IL-6, IL-8, IL-10 and C3-alpha peripheral blood concentrations in single-ventricle patients [164].

### 5.4. Propofol Anesthesia

Propofol is a widely used anesthetic agent working as a ROS scavenger with a chemical structure that resembles vitamin E. Propofol can reduce post-CPB inflammatory markers and lipoperoxidation in adults [165]. In children undergoing CPB for atrial septal defect and VSD repair, the use of propofol resulted in less extubation time after surgery, in addition to a higher serum SOD activity and a lower serum IL-6 concentration during CPB and after cross-clamp removal. Accordingly, less inflammatory cell infiltration and a lower NF-κB expression was observed in myocardial tissue after CPB [166]. In another study that included several complex congenital heart malformations in children, the use of propofol also resulted in lower IL-6, IL-8 and MDA serum concentrations and higher serum SOD activity after CPB [167].

Definite evidence of the participation of oxidative stress in the postoperative clinical evolution of these patients is still lacking, but the available pathophysiological evidence makes it an attractive therapeutic target. Overall, antioxidant-based strategies have not still been properly explored in CPB-induced myocardial and multiorgan dysfunction in children.

## 6. Novel Experimental Antioxidant-Based Therapies

Many of the attempts in modulating oxidative stress in several disease models have been futile. The majority of the tested strategies have been based in antioxidant reinforcement by means of antioxidant supplementation. A general theoretical explanation for the failure of these treatments could be non-selective ROS modulation, which may interfere with physiological ROS-dependent signaling pathways [168], or might not be sufficiently effective in the required cellular type or sub-cellular compartment. Conversely, directed redox modulation could be of more success. Several alternative experimental approaches are being developed following this line of thought. Activation of the Nrf2 pathway by derivatives of fumaric acid can result in an antioxidant effect [169]. Targeting ROS-producing enzymes such as Nox ([170], and myeloperoxidase (MPO) [171], may also result in a more selective ROS modulation in pathologic conditions. An even more innovative approach could be to treat the consequences of the oxidative damage, by regaining loss of enzyme function. As an example, drugs with the potential to prevent or revert ROS-induced eNOS uncoupling might be promising in oxidative stress-related diseases [172]. As discussed, oxidative stress is believed to be a part of the pathogenesis of conditions that may require a much more selective approach, such as neurodegenerative diseases. As an example, modulation of the expression of antioxidant enzymes by using viral-delivery gene therapy may prove to be useful in conditions in which a definite cellular type is identified as target [173]. A highly promising field in experimental medicine is the development of cell therapy, and cardiovascular diseases are no exception [174]. Even in this type of approach, antioxidant-based therapies can be of relevance. Stem cells can be preconditioned to exert an antioxidant effect in target tissues, this way improving their viability and working as a vessel for directed antioxidant delivery [175]. Overall, several innovative antioxidant-based strategies are being developed, but their application in the clinical management of cardiovascular diseases still needs to be clarified.

## 7. Concluding Remarks

A continuously growing body of evidence shows that OS seems to be of key importance in the pathogenesis of several types of cardiovascular diseases. Accordingly, its modulation looks highly attractive from a therapeutic standpoint. Many of the myocardial injuries, such as those seen in ischemia–reperfusion, pharmacologic cardiotoxicity and congenital heart disease surgical correction, are relatively predictable, which offers a unique opportunity for the design of preventive or timely initiated antioxidant-based strategies. These interventions can be as simple as the use of controlled oxygen concentration in CPB or the administration of a specific type of anesthetic, or as complex as the design of multiple-drug protocols. Among the most relevant agents currently being evaluated, omega 3 polyunsaturated fatty acids are promising agents for ischemia preconditioning and anthracycline cardiotoxicity, and carvedilol is a unique beta-blocker with antioxidant properties, besides its role as a first-line heart failure drug. Also, miRNAs are starting to be explored in cardiovascular disease therapeutics. However, proper design clinical trials are still scarce in many of these diseases. It appears that a long road is still ahead before the clinical utility and proper treatment schemes of these agents are properly defined but, so far, the application of redox-based therapeutics in cardiovascular diseases seems most auspicious.

## Figures and Tables

**Table 1:** More important clinical trials with antioxidant therapies in cardiovascular pathologies related with oxidative stress.

Trial (N)	Primary End Point	Treatment/Results (R)	Reference
Kalay et al., 2006 (*n* = 50)	Reduction in LVEF between baseline and 6 months	**Treatment:** Carvedilol 12.5 mg daily vs. placebo. The interventions were initiated prior to the start of chemotherapy and maintained for 6 months. **Results:** Placebo: LVEF 68.9%→52.3%, statistically significant reduction (*p* < 0.001); Carvedilol: LVEF 70.5%→69.7%, no statistically significant reduction (*p* = 0.3)	[129]
OVERCOME Trial (*n* = 90)	The primary efficacy endpoint was the absolute change in LVEF between baseline and 6 months	**Treatment:** Enalapril + carvedilol vs. no treatment Medications titrated as tolerated. Medications started within 1 week before the first chemotherapy cycle and continued for 6 months. **Results:** Control: LVEF 64.6%→57.9%, statistically significant reduction, resulting in a −3.1% absolute difference by echocardiography and −3.4% by cardiac magnetic resonance. Enalapril + carvedilol: LVEF 63.3%→62.9%, no statistically significant changes.	[130]
POAF, Chilean Trial (*n* = 203)	Relative risk of reduction the occurrence of electrocardiographically confirmed POAF from surgery until hospital discharge. Follow-up 14 days.	Patients were randomized to placebo or supplementation with n-3 polyunsaturated fatty acids (2 g/day) (EPA: DHA ratio 1:2), vitamin C (1 g/day), and vitamin E (400 IU/day). **Results:** Supplemented group versus placebo group (relative risk (RR): 0.28) (*p* < 0.01).	[144]
OPERA Trial (*n* = 564)	Incident POAF lasting ≥30 s, centrally adjudicated, and confirmed by rhythm strip or electrocardiography	Fish oil or placebo supplementation (10 g over 3 to 5 days, or 8 g over 2 days). **R:** neither higher habitual circulating omega 3 levels, nor achieved levels or changes following short-term fish oil supplementation are associated with risk of POAF.	[145]
The OMEGA-Study in Critical Ill Patients (*n* = 272)	Patients with acute lung injury would increase ventilator-free days to study day 28.	Twice-daily enteral supplementation of n-3 fatty acids, γ-linolenic acid, and antioxidants compared with an isocaloric control. **R:** patients receiving the omega 3 supplement had fewer ventilator-free days (14.0 vs. 17.2; *p* = 0.02) (difference, −3.2 (95% CI, −5.8 to −0.7)) and intensive care unit-free days (14.0 vs. 16.7; *p* = 0.04). The study was stopped	[146]

LVEF, left ventricular ejection fraction; MI, myocardial infarction; EPA, eicosapentaenoic acid; POAF, postoperative atrial fibrillation; DHA, docosaexaenoic acid.
